# Supplementary material for: Mobility Gaps of Hydrogenated Amorphous Silicon Related to Hydrogen Concentration and Its Influence on Electrical Performance
Source: Nanomaterials (Basel). 2024 Sep 25;14(19):1551. doi: 10.3390/nano14191551 (PMC11477895; doi:10.3390/nano14191551)
Supplement: Supplementary file 1 [file nanomaterials-14-01551-s001.zip › nanomaterials-3163202-supplementary.pdf]

Supporting Information

Mobility Gaps of Hydrogenated Amorphous Silicon Related to Hydrogen Concentration and Its Influence on Electrical Performance

SI_1. Materials and Methods

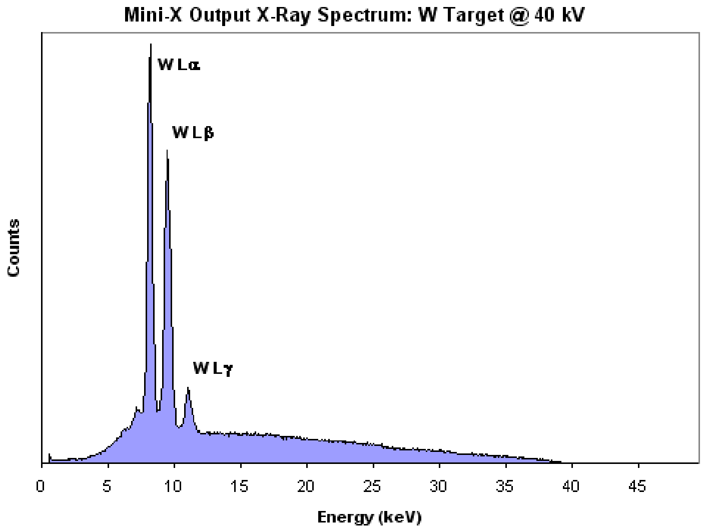

Figure S1. The X-ray tube spectrum at the INFN Perugia laboratory, which was used as the source for the measurements.

Raman spectra were measured at the GHOST laboratory of the University of Perugia using a RM-Horiba iHR320-Triax Raman spectrometer. The details of the experimental setup are reported elsewhere [https://doi.org/10.1038/lsa.2017.139]. The laser spot size is 2 mm.

Here, in brief, we report the main experimental settings used in the presented case. The measurements are performed at room temperature using a single-mode diode-pumped solid-state Spectra-Physics Excelsior operating at 532 nm.

An infinity-corrected apochromatic objective Mitutoyo M-Plan Apo 20× with a very long working distance of 20 mm, and a numerical aperture of 0.42, was used to both focalize and collect the back-scattered light. Due to the high sensitivity of the samples to the laser light, the laser intensity was reduced using neutral optical filters from 3 to 30 mW to avoid any photo-induced alterations on the sample structure [I. Abdulhalim, R. Beserman, and R. Weil, Structural changes and crystallization of amorphous hydrogenated silicon generated by laser irradiation, Phys. Rev. B 39, 1081 - 1091 (1989)].

The sample stability under the chosen illumination conditions was tested before each acquisition.

The depolarized Raman spectra presented herein were acquired using the 600 grooves mm^{-1} grating, which allows the simultaneous acquisition of a wide spectral range from 550 to 3500 cm^{-1} with a resolution of ~10 cm^{-1}. The frequency shifts were calibrated using the crystalline silicon line (520 cm-1) and the background was subtracted by the sp-line.

Photoemission measurements were performed using the BACH beamline at the Elettra synchrotron radiation facility (Trieste, Italy). The beamline is equipped with a hemispherical electron energy analyzer (Scienta R3000) placed at an angle of 60° with respect to the X-ray incident direction. The spectra were collected at a take-off angle of 90° using a photon energy of 1210 eV. The total energy resolution was set to 0.5 eV. The binding energies are referenced against Au $4f_{7/2}$ (84.0 eV) of a gold foil in good electrical contact with the samples. Voigt line shapes and a linear background were used to fit the S 2p spectra. The S $2p_{3/2}$ – S $2p_{1/2}$ spin–orbit splitting and the Lorentzian width at full width at half maximum were fixed at 0.6 eV and 0.1 eV, respectively. The photon beam spot size is in the order of 100 mm.

Inverse photoemission spectroscopy (IPES) measurements were performed in Normal Incidence by using a homemade Erdman–Zipf electron gun. Photons emitted from the sample surface were collected by a homemade Geiger–Mueller-type detector with a He-I2 gas mixture and an SrF2 entrance window filtering photons at hν = 9.5 eV energy. The experimental resolution is better than 300 meV, as measured by the Fermi level onset of a clean Ta foil. Spectra were normalized to the incident electron beam current.

The electron beam divergence is better than 3°; the impinging electron beam spot size is about 1mm. The current density was below 3 10−6 A/cm ^{2}. To control the degradation of the a-Si:H film during measurements, Raman spectra of the samples were measured before and after IPES acquisition.

Different areas of the samples were systematically measured at every spectroscopic characterization to test possible in-plane inhomogeneities of the samples. No detectable differences have been found.

To find a connection between electrical behavior at different operating conditions and spectroscopic characterization, a suitable Technology CAD (TCAD) design methodology can be applied.

The main parameters considered are defect energy level, trap density/occupancy, and Fermi's level; all these parameters are necessary to model this type of detector, built with amorphous material, and are obtained through microscopic measurements.

SI-2 Comparison of Si2p

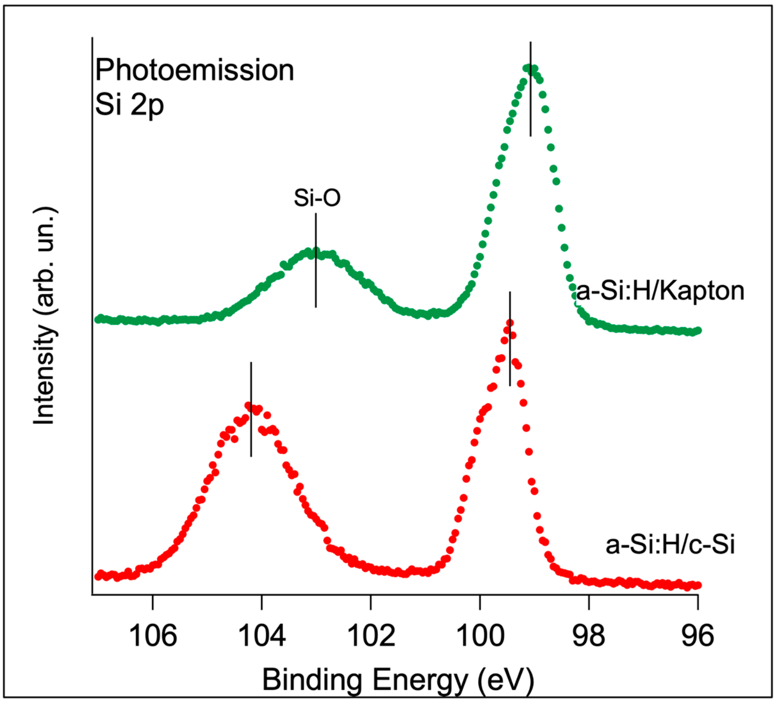

Figure S2. SI-2 Si 2p photoemission ta Si 2p hv=1200 eV of the Kapton (green) and c-Si samples (red).

[1] Zangrando, M.; Zacchigna, M.; Finazzi, M.; Cocco, D.; Rochow, R.; Parmigiani, F. Polarized high-brilliance and high-resolution soft x-ray source at ELETTRA: The performance of beamline BACH. Rev. Sci. Instrum. 2004, 75, 31–36.
